# Digital transformation and corporate tax avoidance: An analysis based on multiple perspectives and mechanisms

**DOI:** 10.1371/journal.pone.0310241

**Published:** 2024-09-18

**Authors:** Qi Zhang, Jinghuai She

**Affiliations:** College of Business Administration, Capital University of Economics and Business, Beijing, China; Shandong University of Science and Technology, CHINA

## Abstract

Promoting the seamless integration of the digital economy with the real economy, mitigating the adverse impacts of widespread corporate tax avoidance, and optimizing tax governance are critical imperatives in the era of digital economy. This study examines all A-share listed companies from 2007 to 2022 as research samples. It utilizes multiple perspectives including signal theory, information asymmetry theory, and the T-O-E (Technology-Organisation-Environment) framework to investigate the primary impacts of digital transformation on corporate tax avoidance, along with the intermediate mechanisms and foundational conditions that influence its effectiveness. After conducting both theoretical and empirical analyses, this paper presents the following conclusions. (1) The implementation of digital transformation significantly reduces corporate tax avoidance, a conclusion supported by rigorous robustness tests. Moreover, digital transformation enhances corporate productivity through the suppression of tax avoidance. (2) Digital transformation diminishes corporate tax avoidance through enhanced innovation and efficiency in resource allocation (technology level), improved quality of internal controls (organization level), and decreased industry competition (environment level). (3) The impact of digital transformation in reducing tax avoidance is significantly greater for enterprises in their growth phase, experiencing lower financing constraints, particularly those situated in the central and western regions. (4) Lower business risk is essential for maximizing the effectiveness of digital transformation and reducing corporate tax avoidance. This is crucial for governments seeking to improve tax administration, guide market and regional development, and enhance the impact of corporate digital transformation on mitigating tax avoidance.

## 1 Introduction

In recent years, the digital economy, supported by digital technologies and characterized by data resources as critical elements, has demonstrated booming developmental trends. It is increasingly emerging as a central force in reshaping national economic competitiveness and the global competitive landscape, guiding a new wave of global economic transformation and development. The “Global Digital Economy White Paper (2023),” published by the China Academy of Information and Communications Technology (CAICT), indicates that in 2022, the estimated added value of the digital economy in 51 countries reached $41.4 trillion. This represents a nominal year-on-year growth of 7.4%, constituting 46.1% of their respective GDP. The rapid ascent of the digital economy has greatly enhanced the exchange of information among market participants. The role of data is becoming increasingly prominent, signaling a fundamental shift in economic paradigms (Stiglitz 2002) [1]. Promoting the profound integration of the digital economy and the real economy has garnered global consensus.

Taxation, as an manifestation of national public power, serves as a crucial revenue source for the state to raise fiscal funds and coordinate economic operations (Wang et al. 2023) [2]. Concurrently, taxation represents a legal obligation and significant cost for enterprises as taxpayers, directly influencing their cash flow and profit scale (Guan et al. 2022) [3]. The implementation of various tax avoidance behaviors by corporates to minimize tax obligations has become a widespread phenomenon globally (Hanlon et al. 2010) [4]. Enterprises employ tax avoidance strategies to redirect resources that would otherwise be diverted to the state for their own development, thereby reducing their cash outflows and boosting net profits (Rego et al. 2012; Zhou et al. 2021) [5, 6]. In recent years, some enterprises have employed tax avoidance measures that skirt the edges of legality, and have even seen frequent cases of tax evasion, significantly impacting the future value of enterprises, increasing the government’s fiscal burden, and hindering the healthy development of capital markets (Xiong et al. 2019; Ye et al. 2014; Yu 2021) [7–9]. The digital economy diverges from traditional business models by ushering in a revolution in production factors and technologies distinct from those of conventional economic models. The concealment and fluidity nature of data elements have disrupted the traditional taxation model that relies on invoice control. This disruption frequently leads to uncertainties about taxable entities and ambiguous tax boundaries (Huang 2023) [10], contributing to challenges such as tax base erosion and issues resembling double taxation in tax administrations (Wang 2020)(Wang 2020) [11]. Consequently, corporate tax avoidance has emerged as a significant issue in tax governance amidst the digital economy era.

Under the surge of the digital economy wave, digitization has evolved into an indispensable skill for corporate development pathways. Digital transformation is systematically restructuring and transforming traditional business logics such as corporate governance structures, business processes, and operational models (Qi et al. 2020) [12], which has had a profound impact on corporate behavior decision making (Wu 2023) [13]. Tax avoidance is essentially the outcome of businesses balancing costs and benefits, and has become one of the crucial decisions in corporate management processes (Hanlon et al. 2010; Badertscher et al. 2013) [4, 14]. Furthermore, digital transformation emphasizes the capability of enterprises to efficiently process and utilize information. If businesses possess a stronger information advantage over government tax regulatory departments, it implies an increased likelihood of concealing transaction information, thereby making it challenging for national tax authorities to effectively regulate and identify tax avoidance activities conducted using digital technologies by enterprises (Li et al.2022) [15]. Zhang et al. (2022) [16] contend that digital transformation exacerbates corporate tax avoidance through increased related-party transactions. However, other studies also suggest that digital transformation can enhance information transparency, thereby restraining corporate tax avoidance (Zhang et al. 2022) [17]. The discussion regarding the relationship between digital transformation and corporate tax avoidance remains inconclusive to date.

The necessity and urgency of optimizing tax governance in the digital economy era have garnered consensus within political and academic spheres (Niu et al. 2023) [18]. Facilitating this consensus, it is crucial to “clarify the core impacts, specific operational mechanisms, and long-term economic consequences of digital transformation on corporate tax avoidance behaviors, thereby providing valuable insights for enhancing tax governance in the digital economy era.” To achieve this, this study selected all A-share listed companies in China from 2007 to 2022 as research samples, and conducted theoretical and empirical examinations based on signal theory, asymmetric information theory, and the T-O-E framework, etc.

The potential marginal contribution of this study lies in: (1) further enriching and refining the study of the relationship between digital transformation and corporate tax avoidance. Existing research on this relationship remains unclear. Zhang et al. (2022) [16] argue that digital transformation exacerbates corporate tax avoidance by increasing related-party transactions; whereas Zhang et al. (2022) [17] suggest that digital transformation suppresses corporate tax avoidance by enhancing corporate transparency. Thus, this study utilizes data from all A-share listed companies to objectively validate the relationship between digital transformation and corporate tax avoidance, thereby extending and complementing existing research (2) This study further enriches the mechanism research on how digital transformation suppresses corporate tax avoidance behaviors. Existing research primarily explores the relationship between digital transformation and tax avoidance from a singular governance perspective focused on internal control quality (Guo, 2023; Zhou, 2022) [19, 20], with scant integrated analyses across technological, organizational, and environmental dimensions. Digital transformation represents an emerging economic development model that systematically restructures and transforms traditional business logics such as corporate governance structures, business processes, and operational models (Qi et al. 2020) [12], exerting significant impacts on organizational management, environments, and technological advancements. The comprehensive organizational changes also further influence corporate tax avoidance decisions. Therefore, this paper, based on the T-O-E framework and a systemic perspective, investigates the intermediate mechanisms through which digital transformation influences corporate tax avoidance, thereby opening up the black box of its multiple mechanisms and offering additional developmental insights to leverage the practical effectiveness of digital transformation. (3) This study further enriches the perspective on the relationship between digital transformation and corporate tax avoidance. Previous research has rarely considered the impact of business risks on the relationship between digital transformation and tax avoidance. This paper integrates business risks into the research framework, exploring how digital transformation affects the effectiveness of corporate tax avoidance from a risk management perspective. This contributes theoretical and practical guidance for businesses to empower sustainable development through digital transformation.

## 2 Theoretical foundation and research hypotheses

### 2.1 Theoretical foundation

Corporate tax avoidance refers to all activities that definitively reduce the tax burden of enterprises, encompassing both completely legal and those in a gray area (Dyreng et al. 2008) [21]. Minimizing tax expenditures to maximize corporate profits has become a common practice among managers. Through tax avoidance practices, corporate managers can balance non-tax costs and benefits, manipulate financial information (Kim et al. 2011) [22]; and consequently alter corporate value and operational performance (Desai et al. 2006) [23]. Companies often engage in tax avoidance through methods such as income concealment, expense inflation, and transfer pricing (Yu et al. 2022) [24]. This brings significant potential costs to enterprises; if tax avoidance is detected and investigated by tax authorities, companies may need to pay back taxes and substantial fines (Rego et al. 2012) [5]. Tax avoidance retains a large portion of corporate funds internally but only serves short-term interests (Weisbach 2002) [25]. Moreover, as an opportunistic behavior, tax avoidance significantly reduces government tax revenue, distorts resource allocation, and undermines national tax security (Chen et al. 2023) [26]. The academic and practical communities have long been devoted to exploring the factors influencing corporate tax avoidance. Externally, factors such as tax enforcement intensity (Dubin et al. 1990; Fan et al. 2013) [27, 28], government policy uncertainty (Chen et al. 2016) [29], industrial policies (Zhang et al. 2021) [30], openness of capital markets (Zhi et al. 2021) [31], financing environment (Edwards et al. 2016) [32], labour markets (Kubrick et al. 2016) [33], geographical location (Chen et al. 2022) [34], and stakeholders such as suppliers and customers (Cen et al. 2017) [35] can all influence corporate tax avoidance behaviors. Internally, factors including managerial capabilities (Dai et al. 2016) [36], corporate governance and ownership structure (McGuire et al. 2014) [37], internal controls (Zhou 2019) [38], and board characteristics (Armstrong et al. 2015) [39], directly influence corporate tax avoidance behaviors. For instance, Kovermann et al. (2019) [40] research found that corporate governance structures not only potentially increase corporate tax avoidance behaviors but also constrain tax avoidance risks below to benefits. There is considerable debate about the factors influencing corporate tax avoidance behaviors, with few studies integrating the characteristics of the digital economy era to explore these factors comprehensively.

In recent years, scholars have also begun to recognize the significance of the digital economy on corporate tax avoidance behaviors. In the era of digital economy, digital transformation has become an inevitable choice for enterprises to adapt to the trends of the times and pursue long-term development. Existing studies suggest that digital transformation leverages digital technologies to drive organizational change (Bharadwaj et al. 2013) [41], significantly enhancing enterprises’ capabilities in information acquisition and processing, reducing information asymmetry, and consequently lowering audit costs (Zhang et al. 2021) [42]. It also improves productivity and innovation efficiency (Loebbecke et al. 2015; Chen et al. 2020) [43, 44]. Moreover, it enhances stock liquidity (Wu et al. 2021) [45], elevates corporate governance and internal control quality (Qi et al. 2020; Zhang et al. 2022) [46, 47], fosters innovation in business models, and creates additional value (Fitzgerald et al. 2014; Nambisan et al. 2019; Bouwman et al. 2019) [48–50]. Consequently, it increases corporate cash holdings and reduces occurrences of corporate tax avoidance decisions (Frischmann et al. 2008) [51]. However, due to the disruptive nature of digitalization on traditional models (Vial 2019) [52], traditional tax regimes find it challenging to timely adapt to the pace of digital economic development (Gu et al. 2022) [53], thus increasing the difficulty of tax supervision and posing significant challenges to China’s existing tax management system (Hu et al. 2019) [54]. Simultaneously, the ambiguous definition of the digital economy scope and the virtual nature of digital transactions make it difficult to accurately define taxpayers, transaction behaviors, and value creation (Niu et al. 2023; Li et al. 2022; Ma et al. 2021) [18, 55, 56], facilitating profit shifting (Wang et al. 2020) [11] and thereby increasing the potential for corporate tax avoidance.

The aforementioned studies have recognized the urgency and necessity of optimizing tax governance in the digital economy era, and have attempted to explore the relationship between digital transformation and corporate tax avoidance at the micro-enterprise level, thereby offering theoretical and practical references for improving tax governance in the digital economy. However, existing research is still in its infancy, lacking further theoretical and empirical studies on the relationship between digital transformation and corporate tax avoidance. Furthermore, discussions in existing studies regarding this relationship remain inconclusive, characterized by significant ambiguity. Lastly, there is a scarcity of studies employing systematic theory to provide comprehensive empirical evidence on the relationship between digital transformation and corporate tax avoidance. Therefore, the above analysis also provides substantial theoretical and empirical space for the development of this study.

The theory of information asymmetry posits that asymmetry in information between taxpayers and tax authorities pose significant challenges in tax administration. Effective information flow plays a crucial role in achieving tax governance (Pomeranz 2015) [57]. The generation of corporate tax avoidance behavior largely depends on the degree of information asymmetry. Signal theory suggests that enterprises engage in digital transformation, utilizing digital technologies to enhance their capabilities in information acquisition and processing (Loebbecke et al. 2015) [43], deeply integrating into internal governance processes. Furthermore, compared to external markets, enterprises can act as the advantaged party in digital transformation information, promptly acquiring more information. This promotes continuous evolution of top-down internal governance paradigms, enhances corporate transparency and information flow efficiency, increases the likelihood of detecting corporate tax avoidance behaviors, reduces tax administration difficulties, and raises the costs and risks associated with tax avoidance (Kerr 2019) [58]. Simultaneously, it effectively curbs management’s motives for tax avoidance rent-seeking and mitigates principal-agent problems (Wang et al. 2023) [2], thereby creating conditions to mitigate corporate tax avoidance issues. However, digital transformation is not merely about simplistic technological applications; it represents a long-term, high-risk organizational change activity (Hanelt et al. 2021) [59]. This process, akin to playing a “symphony” (AlNuaimi et al. 2022) [60], represents the result of comprehensive changes in products, services, business processes, business models, organizational structures, and organizational culture, posing significant challenges to enterprise strategy implementation and formulation (Yu et al. 2024) [61]. At the same time, tax avoidance, as a set of tax planning decisions for enterprises to reduce tax obligations and lessen tax burdens (Dyreng et al. 2010; García-Meca et al., 2021) [62, 63], is significantly influenced by digital transformation, reflecting the outcome of comprehensive organizational change. Therefore, this study will conduct a logical analysis of how digital transformation affects corporate tax avoidance, based on the T-O-E framework, focusing on technological, organizational, and environmental dimensions.

### 2.2 Research hypotheses

Digital transformation represents a thoroughgoing organizational change process initiated from the top-down (Vial 2019) [52], which is anticipated to profoundly impact the technological, organizational, and environmental dimensions within organizations, thereby influencing the formulation of corporate tax avoidance strategies (Zhang et al. 2022) [17].

#### A. Technology level

Digital transformation, driven by digital technologies, is fundamentally reshaping economic and social production as well as lifestyles, innovating beyond traditional paradigms. It can leverages the catalytic effects of data elements, providing greater support for enterprise research and development innovation (Dang et al. 2021) [64], thereby encouraging enterprises to increase their investment in research and development. Endogenous growth theory posits that innovation optimizes the integration of knowledge, technology, and other production factors, leading to sustained positive excess returns for enterprises (Cohen et al. 2013) [65]. This increases enterprise cash flows and consequently diminishes tax avoidance motives. Furthermore, digital transformation improves the allocation of production factors in enterprises through technological upgrades, facilitating the rational distribution of resources (Zhao Chenyu et al. 2021) [66], creating conditions for enterprises to fulfill tax obligations, and thereby reducing aggressive tax avoidance behaviors.

From a behavioral perspective, digital transformation leverages modern information technology tools to firstly overall promote enterprises to overcome innovation dilemmas (Zhuo et al. 2023) [67], and improve the innovation ecosystem. Digital technology disrupts traditional innovation processes by embedding traditional production factors or conditions, making innovation increasingly reliant on complex digital changes. Secondly, digital transformation empowers innovation by providing more information, material, and human resources (Ling et al. 2024) [68], stimulating innovation. Additionally, digital transformation also possesses collaborative functions, efficiently capturing and processing various diverse and complex information, rapidly seizing innovation opportunities (Rialti et al. 2018; Tan et al. 2015) [69, 70], strengthening the coupling and correlation of enterprise information networks, promoting the transformation of data elements into infinitely reusable new knowledge (Cong et al. 2021) [71], reducing information asymmetry and costs(Song et al. 2022) [72], and encouraging enterprises to increase innovation investment (Wang et al. 2024) [73]. Corporate innovation is a manifestation of its pursuit of long-term excess returns. Companies with higher investment in innovation have a positive announcement effect, bringing positive excess returns to enterprises (Cohen et al. 2013) [65]. At the same time, it can also conserve cash flow for enterprises, shield enterprises from cash flow shocks, and thus reduce the occurrence of enterprises retaining cash through tax avoidance behaviors (Rego et al. 2012) [5].

From a performance perspective, according to organizational information processing theory, digital transformation addresses complex information changes, accelerates information flow, and transforms both internal and external enterprise environments. Firstly, digital technologies further enhance information transparency (Guan et al. 2022) [3], helping enterprises grasp information advantages swiftly, reducing information search costs, and accurately identifying market investment opportunities (Yan et al. 2023) [74]. Secondly, digital transformation strengthens internal connections, reduces internal information exchange costs, empowers enterprises with precise control over internal resource elements, facilitates deep integration of internal and external market resources, and accelerates the flow of production factors within enterprises (Zhang et al. 2021) [75]. Additionally, digitalization promotes cross-regional flow of production factors, enhances linkage among customers, enterprises, and suppliers at various stages, and improves efficiency in supply chain operations (Li et al. 2023) [76]. The improvement in information transparency, enhanced internal information linkage, and increased efficiency in supply chain operations will further enhance enterprise resource allocation efficiency. Restuccia’s study (2017) [77] indicates that inefficiencies in resource allocation can lead to significant losses in production efficiency, resulting not only in economic losses but also heightening management’s expectations of risk, including potential tax risks. Therefore, it will further stimulate enterprise risk management awareness, spur tax avoidance, aiming to capitalize on tax avoidance to generate revenue for enterprises, thereby maximizing private profits.

#### B. Organisation level

The quality of internal controls is the most direct reflection of organizational management effectiveness within enterprises. The enhancement of internal control levels holds profound significance in strengthening internal oversight and optimizing corporate governance (Wang et al. 2023) [2]. Against the backdrop of digitalization, enterprises integrate fragmented information to promote interconnectedness across all stages. Work environments and business processes are gradually institutionalized and transparent, shaping organizational structures towards increased flatness and grid-like configurations (Qi et al. 2020) [12]. Organizational management shifts from centralized to decentralized management (Adner et al. 2019) [78], thereby establishing a scientific system of rights and responsibilities allocation, enhancing internal control effectiveness. This transition further prevents management from overriding internal controls (Wu 2023) [13], strengthens internal oversight, refines internal supervision and punitive mechanisms, and curbs aggressive tax avoidance behaviors (Chen et al. 2015) [79]. Furthermore, digital transformation enhances the accessibility and transparency of information acquisition, ensuring timely and accurate internal communication within enterprises, and boosting information flow efficiency (Nie et al. 2022) [80], Consequently, it also renders tax-related matters more open and transparent, fostering trust from external tax authorities towards enterprises, promoting compliant business operations, and thereby effectively prevents the occurrence of tax avoidance behaviors (Yu et al. 2021) [81]. Additionally, digital transformation benefits comprehensive enhancement of internal control tracking efficiency and strengthens detection sensitivity (Luo et al. 2021) [82], and drives intelligent transformation of internal control systems, effectively balances the interests among stakeholders, improves the quality of internal control information disclosure, assists tax authorities and other regulatory agencies in more effectively assessing enterprises’ tax compliance, thereby increasing the risk of detecting and penalizing tax avoidance behaviors, and consequently reducing occurrences of tax avoidance.

#### C. Environment level

The abundant accumulation of resources brought about by a relaxed competitive environment often makes enterprises within such environments more inclined to follow the guidance of the institutional environment (Sun et al. 2021) [83]. In the era of digital economy, enterprises are immersed in a normalized environment of uncertainty (Wang et al. 2023) [84]. The uncertainty of the environment exacerbates the unpredictable turbulence within industries and markets at the micro level, triggering fluctuations in business revenues and other performance metrics (Ghosh et al. 2009) [85]. Simultaneously, environmental uncertainty leads to frequent policy adjustments, making it difficult for enterprises to adapt promptly, resulting in decision-making errors. Consequently, this may trigger corporate risk management and earnings management mechanisms, reinforcing the incentive for enterprises to generate revenue through tax avoidance. Digital transformation has demonstrated the undeniable advantages in enhancing information connectivity and imporving information transparency (Qi et al. 2020; Nie et al. 2022) [12, 80]. It facilitates effective transmission of information both within and outside enterprises, reducing information discrepancies between internal and external decision-makers. This also enhances the accuracy of assessing each other’s behaviors, enabling timely and correct decision-making, thereby collectively fostering a favorable market environment, standardizing corporate behaviors within the current market context, enhancing compliance with market regulations by enterprises, and reducing the emergence of aggressive tax avoidance behaviors.

Based on the analysis above, this paper proposes the following research hypotheses:

H1: Controlling for the effects of other factors, digital transformation can significantly suppress the emergence of tax avoidance behaviors.

**Fig 1:**
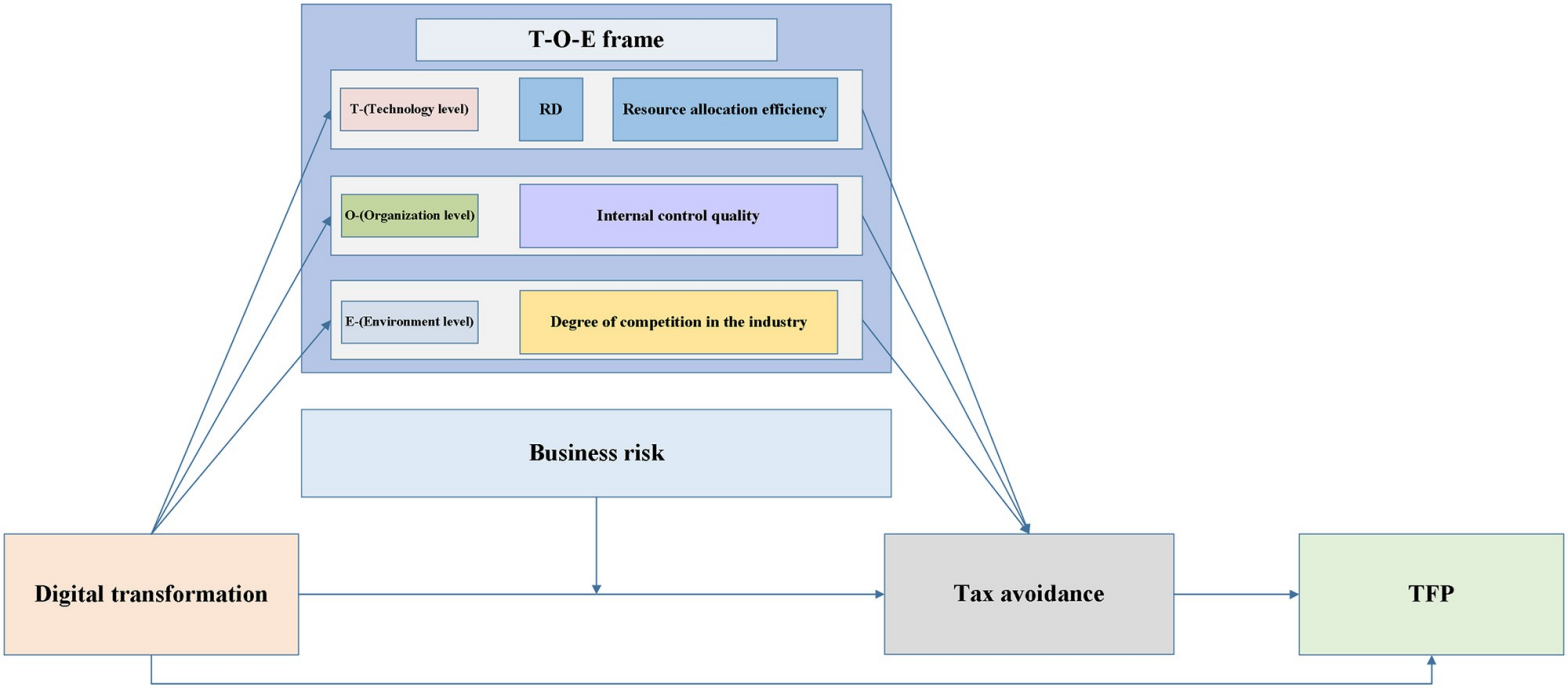
Theoretical model diagram. And the diagram is sourced from the author’s own drawing.

Meanwhile, based on the research background and framework of this study, a model diagram is proposed, as illustrated in Fig 1.

## 3 Data and model

### 3.1 Sample selection and data sources

Considering the impact of the 2006 revision of Chinese accounting standards on the financial data of listed companies, this study selects A-share listed companies on the Shanghai and Shenzhen stock exchanges from 2007 to 2022 as the research sample. To enhance sample comparability, this study implemented the following selection criteria commonly adopted in most existing research: (1) excluding samples from financial and insurance companies; (2) excluding companies that underwent initial public offerings (IPOs) in the same year (typically, companies undergoing IPOs in the same year do not have annual reports); (3) excluding samples of companies designated with special treatments such as ST and PT by stock exchanges; (4) excluding samples of companies with pre-tax profits less than or equal to zero, abnormal actual income tax rates (i.e., rates less than 0 or greater than 1), and income tax expenses less than zero; (5) excluding samples of companies with irreparable financial data gaps. Additionally, this study applied a 1% winsorization to all micro-level continuous variables. The original data primarily come from the CSMAR and Wind database. Annual report data of relevant listed companies are sourced from the official websites of the Shenzhen and Shanghai stock exchanges. Ultimately, 30,845 valid observations were obtained.

### 3.2 Variable definitions

#### 3.2.1 Dependent variable

Degree of tax avoidance (BTD). This study adopts the concept of Book-Tax Differences (BTD) to measure the extent of corporate tax avoidance, following the studies by Desai et al. (2006) and Ye et al. (2014) [8, 23]. Specifically, BTD is defined as BTD = (Pretax Accounting Income—Taxable Income) / Total Assets at the end of the period; where Taxable Income = Current Income Tax Expense / Nominal Income Tax Rate = (Income Tax—Deferred Income Tax Expense) / Nominal Income Tax Rate. A higher BTD indicates greater tax avoidance, and conversely.

#### 3.2.2 Independent variable

Digital transformation (DT). Drawing from Qi et al. (2020) [46] and Zhang et al. (2021) [42], the ratio of year-end intangible assets disclosed in the financial statements’ notes of publicly listed companies, pertaining to digital transformation, relative to total intangible assets, serves as a measure of corporate digitalization level. Specifically, when the detailed items of intangible assets include keywords such as “software,” “network,” “client,” “management system,” “intelligent platform,” and related patents associated with digital transformation technologies, these items are defined as “digital technology intangible assets.” Subsequently, the total of multiple digital technology intangible assets of the same company for the same fiscal year is aggregated, and the proportion to total intangible assets for the year is calculated. The resulting outcome is thereby established as a proxy variable representing the extent of corporate digital transformation, denoted as DT. A higher DT indicates a greater degree of corporate digital transformation, and vice versa.

#### 3.2.3 Control variables

The existing literature extensively employs financial characteristics as control variables when examining corporate tax avoidance (Li 2008) [86]. To enhance research precision, this study follows the methodology of Li et al. (2023) [87] and incorporates the following control variables into the model: firm size (SIZE), leverage ratio (LEV), return on equity (ROE), growth (GROWTH), investment opportunities (BM), prior-year loss (LOSS), firm age (AGE), duality (DUAL), equity concentration (TOP1), fixed asset ratio (FIXED), intangible asset ratio (INTANGIBLE), independent director ratio (INDEP), ownership nature (SOE), Big Four auditing (BIG4), and audit opinion (OPINION). Additionally, this study incorporates firm fixed effects and year fixed effects. To eliminate the interference of diverse tax rates across cities on regression outcomes, it also further incorporates city fixed effects. Lastly, it incorporates high-dimensional year-industry fixed effects to eliminate potential impacts of industry-specific tax rate changes and policy shocks on regression results. Specific variable definitions are detailed in Table 1.

**Table 1:** Definitions of variables.

Type	Variable	Symbol	Measurement
*Dependent variable*	Degree of tax avoidance	BTD	(Pre-tax accounting profit—taxable income) / total assets at the end of the period
*Independent variable*	Digital transformation	DT	The proportion of intangible assets related to digital transformation disclosed in the year-end intangible asset details of the financial statements of listed companies.
*Control variables*	Firm size	SIZE	The natural logarithm of total assets at year-end
	Leverage ratio	LEV	Total liabilities at year-end/total assets at year-end
	Return on equity	ROE	Return on equity of the firm
	Growth	GROWTH	Current year’s operating revenue / previous year’s operating revenue-1
	Investment opportunities	BM	Book value of equity capital / market value
	Prior-year loss	LOSS	If the firm’s net profit for the previous year is negative, the value is 1; otherwise, it is 0.
	Firm age	AGE	Number of years since the company went public, calculated as the current year minus the year of listing plus 1.
	Duality	DUAL	Equal to 1 if the Chairman and the CEO are the same person (DUAL); otherwise, 0.
	Equity concentration	TOP1	Number of shares held by the largest shareholder / total number of shares
	Fixed asset ratio	FIXED	Net fixed assets / total assets
	Intangible asset ratio	INTANGIBLE	Net intangible assets/total assets
	Independent director ratio	INDEP	Proportion of independent directors on the board of directors.
	Ownership nature	SOE	If it’s a state-owned enterprise, the value is 1; otherwise, it’s 0.
	Big Four auditing	BIG4	A value of 1 is assigned if audited by one of the Big Four accounting firms in the given year; otherwise, the value is 0.
	Audit opinion	OPINION	A value of 1 is assigned if the annual report of the listed company receives a non-standard audit opinion; otherwise, the value is 0.

### 3.3 Econometrics model

To investigate whether and how digital transformation affects corporate tax avoidance, this study draws on Chen et al. (2023) [26] to first construct Model (1) to examine the overall impact of digital transformation on corporate tax avoidance.

$$\begin{array}{c} {\text{BTD}}_{i,t}=\beta_{0}+\beta_{1}DT_{i,t}+\sum_{i=1}^{n}Controls_{i,t}+{\text{Firm}}_{i}+Year_{t}+City_{c}+Year_{t}*Industry_{h}+\epsilon_{i,t} \end{array}$$ (1)

In model (1), BTD_i,t_ represents firm i’s extent of tax avoidance in year t. DT_i,t_ represents the level of digital transformation of firm i in year t. Controls_i,t_ is the set of control variables including SIZE, LEV, ROE, GROWTH, BM, LOSS, AGE, DUAL, TOP1, FIXED, INTANGIBLE, INDEP, SOE, BIG4 and OPINION. Firm_i_ represents firm-level fixed effects. Year_t_ represents year fixed effects. City_c_ represents city fixed effects. Year_t_*Industry_h_ represents year-industry high-dimensional fixed effects. And *ϵ*_i,t_ represents the random error term.

## 4 Empirical analysis

### 4.1 Descriptive analysis

Table 2 displays the descriptive statistics of variables and their variance inflation factor (VIF) values. The results indicate that the average tax avoidance level (BTD) among sampled firms in this study is 0.002. On average, the accounting profits of sampled firms exceed their taxable income, suggesting that tax avoidance remains prevalent in the digital economy era. The average degree of digital transformation (DT) among sampled firms is 0.09, with a minimum of 0 and a maximum of 1, indicating significant variability in digital transformation levels among sampled firms, with some yet to undergo digital transformation. The descriptive statistics of other control variables fall within reasonable ranges compared to existing research. Furthermore, VIF values for all variables are below 5, with an average VIF well below 10, indicating no multicollinearity among the variables studied in this research.

**Table 2:** Descriptive statistical results.

Variables	Obs	Mean	Std. Dev.	Min	Max	VIF
*BTD*	30,845	0.002	0.027	-0.07	0.092	
*DT*	30,845	0.090	0.215	0	1	1.11
*SIZE*	30,845	22.24	1.304	19.96	26.31	2.28
*LEV*	30,845	0.421	0.197	0.056	0.855	1.48
*ROE*	30,845	0.1	0.074	0.004	0.391	1.29
*GROWTH*	30,845	0.202	0.399	-0.443	2.607	1.10
*BM*	30,845	0.624	0.247	0.124	1.183	1.66
*LOSS*	30,845	0.0002	0.014	0	1	1.00
*AGE*	30,845	2.122	0.813	0	3.332	1.58
*Dual*	30,845	0.262	0.440	0	1	1.14
*Top*1	30,845	35.35	14.75	9.310	74.89	1.15
*FIXED*	30,845	0.215	0.159	0.003	0.697	1.12
*INTANGIBLE*	30,845	0.046	0.052	0.0001	0.337	1.06
*INDEP*	30,845	37.43	5.331	30.77	57.14	1.03
*SOE*	30,845	0.389	0.487	0	1	1.48
*BIG*4	30,845	0.065	0.246	0	1	1.19
*OPINION*	30,845	0.987	0.112	0	1	1.01
*Mean of VIF*						1.29

Note: All table data in this study are drawn by the author himself. The following text is the same.

In addition, following DT’s annual industry median, this study categorizes sampled enterprises into two groups based on their degree of digital transformation. Table 3 presents the results of univariate analysis for these two groups of samples. The results indicate that, whether through mean t-tests or median Wilcoxon rank-sum tests, there are significant differences in the mean BTD between the groups with high and low degrees of digital transformation. Moreover, compared to enterprises with low digitalization levels, those with high digital transformation exhibit lower levels of tax avoidance. This suggests that digital transformation in enterprises can reduce the degree of tax avoidance, thereby providing preliminary validation for hypotheses 1 in this study.

**Table 3:** Univariate analysis results.

	Mean t-test			Wilcoxon test
Variable	Degree of digital transformation			Z-statistic
	High group	Low group	Mean difference	H0: Equal means for high and low groups
*BTD*	0.001	0.002	-0.001^***^	4.248^***^

Note: The independent sample t-test was used for the mean test; the Wilcoxon rank sum test was used for the median test.

^***^ indicates significant at the 1 per cent statistical level.

### 4.2 Baseline regression

Table 4 reports the baseline regression results on the relationship between “digital transformation—corporate tax avoidance”. To ensure accurate interpretation of the regression model regarding the relationship between the dependent variable BTD and the independent variable DT in this study, panel mixed OLS, fixed effects, and random effects were initially selected for regressing the dependent variable, independent variables, and control variables. Columns (1) to (3) of Table 4 respectively present the regression outcomes using panel data OLS, fixed effects, and random effects. The coefficient of the OLS regression in column (1) is significantly negative at the 5% level, indicating that, after controlling for other relevant influences, digital transformation (DT) indeed reduces corporate tax avoidance. Comparing the results in columns (2) and (3), the regression coefficient estimated using panel fixed effects shows a significant increase compared to that estimated using panel random effects, and the P-value of the Hausman test is far below 0.05. Therefore, the fixed effects model appears more suitable for this study. Considering the effects of individual, time, city, and year-industry heterogeneities, we further employ a high-dimensional fixed effects model for estimation. In column (4) of Table 4, controlling for individual and year fixed effects, the results show that the regression coefficient of DT on BTD is -0.0020, significant at the 5% level. In column (5), after additionally including city and year-industry fixed effects, the regression coefficient of DT on BTD increases to -0.0021 from -0.0020 in column (4), and still passes the 5% statistical significance test. This also indicates the effectiveness of the estimated model in this study. Once again, evidence is provided for the conclusion that digital transformation reduces corporate tax avoidance. Thus, hypotheses 1 is validated.

**Table 4:** Baseline regression results.

	(1)	(2)	(3)	(4)	(5)
	BTD	BTD	BTD	BTD	BTD
	OLS	FE	RE		
*DT*	-0.0017**	-0.0019**	-0.0017**	-0.0020**	-0.0021**
	(-2.15)	(-2.05)	(-2.15)	(-2.01)	(-2.09)
*Control variables*	Yes	Yes	Yes	Yes	Yes
*Firm effect*	No	No	No	Yes	Yes
*Year effect*	No	No	No	Yes	Yes
*City effect*	No	No	No	Yes	Yes
*City effect*	No	No	No	No	Yes
*Year* − *Industry effect*	No	No	No	No	Yes
*Obs*	30,845	30,845	30,405	30,405	30,405
*adj*.*R*^2^				0.431	0.434
*Hausman*	299.91***				

Note: (1) The reduction in sample size after applying the high-dimensional fixed-effects model occurs because the fixed effects cause some observations to become “singleton observations.” The high-dimensional fixed-effects model excludes these observations in the estimation process; however, this does not impact the validity of the study’s conclusions. (2) *** and ** denote significant at the 1% and 5% levels, respectively. (3) Parentheses indicate t-values adjusted by clustering robust standard errors.

### 4.3 Robust tests

#### 4.3.1 Endogeneity test

The results of this study indicate that digital transformation significantly reduces corporate tax avoidance. However, since digital transformation requires substantial investment, and tax avoidance may affect resource allocation, corporate tax avoidance can, in turn, influence digital transformation decisions, leading to a bidirectional causality issue. To address this, we use an instrumental variable approach to mitigate the endogeneity issue in the baseline regression model. Following Ma et al. (2023) [88], we select the mean digital transformation of other firms within the same city and industry (IV1) and the cube of the difference between the firm’s digital transformation and the mean digital transformation by province and industry (IV2) as instrumental variables for the regression analysis. On one hand, the digital transformation levels in the firm’s city, province, and industry affect managers’ digital transformation decisions, satisfying the relevance assumption for the instrumental variables. On the other hand, the level of digital transformation in the firm’s city, province, and industry does not directly affect the firm’s tax avoidance behavior, satisfying the exogeneity assumption for the instruments. Table 5 presents the IV-2SLS regression results. The first stage results in column (1) of Table 5 show that the regression coefficients of IV1 and IV2, 0.317 and 1.796 respectively, are statistically significant at the 1% level, indicating IV1 and IV2 are correlated with the independent variable DT. The second stage results in column (2) of Table 5, show that the Kleibergen-Paap rk LM test rejects the null hypothesis of instrument underidentification at the 1% significance level. The Cragg-Donald Wald F statistic exceeds the critical value of 19.93 for the Stock-Yogo weak instrument F test at the 10% significance level, rejecting the weak instrument null hypothesis. The Hansen J statistic passes the 5% significance test, ruling out over-identification issues. Additionally, the negative correlation between digital transformation (DT) and tax avoidance (BTD) remains significant. Furthermore, the regression coefficient of DT on BTD is greater than that observed in the baseline regression results. This indicates that the baseline regression underestimates the impact of digital transformation on corporate tax avoidance, and the main research conclusion presented earlier remains valid.

**Table 5:** Instrumental variable regression results.

	(1)	(2)
	DT	BTD
	2SLS:First stage	2SLS:Second stage
*DT*		-0.003**
		(-2.33)
*IV*1	0.317***	
	(4.69)	
*IV*2	1.796***	
	(36.79)	
*Control variables*	Yes	Yes
*Firm effect*	Yes	Yes
*Year effect*	Yes	Yes
*City effect*	Yes	Yes
*Year* − *Industry effect*	Yes	Yes
*Obs*	30,303	30,303
*Kleibergen* − *Paap rk LM statistic*		254.701***
*Cragg* − *Donald Wald F statistic*		28000
		[19, 93]
*Hansen J statistic*		5.288**

Note: (1) *** and ** indicate significant at the 1% and 5% levels, respectively. (2) Parentheses denote t-values adjusted for robust standard errors by clustering. (3) The identifiability test was conducted using the K-Paap rk LM statistic; the weak instrumental variable test was conducted using the Cragg-Donald Wald F statistic, and the values in square brackets are the critical values of the F-test for Stock-Yogo’s weak instrumental variables at the 10% level of significance; the over-identification test was conducted using the Hansen J statistic was used for the test.

#### 4.3.2 Sample selection biases

To mitigate the impact of sample selection bias on causal inference, this study employs Propensity Score Matching (PSM) for robustness checks (Rosenbaum et al. 1983) [89]. To ensure the reliability of the PSM results, samples with digital transformation (DT) values above the median are defined as the treatment group. Initially, a matching quality assessment is conducted on the entire sample, and PSM neighbor matching, PSM kernel matching, and PSM radius matching methods are used for comparison during the analysis. Finally, causal inference between the independent variable (DT) and the dependent variable (BTD) is conducted for the matched samples. The results are detailed in Table 6. Columns (1) to (3) of Table 6 show the regression results from PSM neighbor matching, PSM kernel matching, and PSM radius matching, respectively. The regression coefficients of DT on BTD are significantly negative at the 5% significance level, consistent with the baseline regression results.

**Table 6:** PSM and Heckman regression results.

	(1)	(2)	(3)	(4)
	BTD	BTD	BTD	BTD
	PSM:1-1 nearest neighbour matching	PSM:Kernel matching	PSM:Radius matching	Heckman:Second stage
*DT*	-0.004**	-0.002**	-0.002**	-0.002**
	(-2.59)	(-2.06)	(-2.06)	(0.001)
*IMR*				0.317***
				(4.69)
*Control variables*	Yes	Yes	Yes	Yes
*Firm effect*	Yes	Yes	Yes	Yes
*Year effect*	Yes	Yes	Yes	Yes
*City effect*	Yes	Yes	Yes	Yes
*Individual effect*	Yes	Yes	Yes	Yes
*Year* − *Industry effect*	Yes	Yes	Yes	Yes
*Obs*	15,511	30,395	30,395	17,024
*adj*.*R*^2^	0.428	0.434	0.434	0.499

Note: (1) ***, **, and * indicate significant at the 1%, 5%, and 10% levels, respectively. (2) Parentheses indicate t-values adjusted by clustering robust standard errors.

#### 4.3.3 Sample self-selection effect

Given that digital transformation and tax avoidance are managerial decisions made subjectively by managers during the firm’s development process, there may be sample self-selection bias affecting Model (1). To address this issue, the study employs the Heckman two-step method (treatment effect model) for robustness checks to mitigate the impact of self-selection bias on the regression results. First, the sample firms are divided into high and low digitalization groups based on the average digital transformation value, creating a new variable, DTdum. If a firm is in the high digitalization group, set DTdum to 1; otherwise, set it to 0. Next, using the control variables from the baseline regression, a probit regression is conducted to calculate the Inverse Mills Ratio (IMR). Finally, IMR values are included in Model (1) for re-estimation. Column (4) of Table 6 reports the regression results from the second stage of the Heckman two-step method. The IMR coefficient is significantly positive, indicating the appropriateness of the Heckman two-step model. The coefficient of digital transformation (DT) on BTD remains significantly negative at -0.002, with no substantial change in magnitude. This indicates that, after addressing sample self-selection bias in the baseline model using the Heckman two-step method, the conclusion that digital transformation significantly inhibits tax avoidance behavior remains valid.

#### 4.3.4 Other robustness tests

To further ensure the robustness of the research findings, additional robustness tests were conducted.

First, the measurement of the core variable was altered. Following the approach of Guan et al. (2022) [3], digital transformation was treated as a binary variable, assigning a value of 1 if the firm underwent digital transformation and 0 otherwise. This new variable, DT1, was used as an alternative measure of digital transformation DT in the regression analysis. Additionally, following Ye et al. (2014) [8], the impact of accrual-based earnings management was further removed by using accounting-tax differences (DDBTD) as an alternative measure for the dependent variable BTD. Columns (2)-(3) in Table 7 display the regression results with the substituted independent variable and dependent variables. The regression coefficients of digital transformation DT on BTD remain significantly negative at the 5% and 10% statistical levels, consistent with the baseline regression results. Thus, whether substituting the independent variable or the dependent variable, the model’s accuracy is statistically validated.

**Table 7:** Other robustness test regression results.

	(1)	(2)	(3)	(4)	(5)	(6)
	BTD	BTD	DDBTD	BTD	BTD	BTD
*DT*			-0.003***	-0.002*	-0.002**	-0.005***
			(-2.79)	(-1.89)	(-2.07)	(-3.80)
*DT*1		-0.001**				
		(-2.56)				
*L*2.*DT*	-0.003**					
	(-2.02)					
*Control variables*	Yes	Yes	Yes	Yes	Yes	Yes
*Firm effect*	Yes	Yes	Yes	Yes	Yes	Yes
*Year effect*	Yes	Yes	Yes	Yes	Yes	Yes
*City effect*	Yes	Yes	Yes	Yes	Yes	Yes
*Year* − *Industry effect*	Yes	Yes	Yes	Yes	Yes	Yes
*Obs*	20,725	30,845	30,405	24,103	30,259	13,070
*adj*.*R*^2^	0.465	0.434	0.441	0.451	0.436	0.455

Note: (1) ***, **, and * indicate significant at the 1%, 5%, and 10% levels, respectively. (2) Parentheses indicate t-values adjusted by clustering robust standard errors.

Second, changing the sample interval. (1) Corporate activities are often influenced by external environmental factors (Tang et al. 2020; Wu et al.,2021) [45, 90]. Given the significant impacts of the 2007 corporate income tax reform, the 2008 financial crisis, the 2015 Chinese stock market crash, and the 2020 COVID-19 pandemic on economic development and their potential effects on enterprises, samples from 2007, 2008, 2015, and 2020 were excluded and the regression was re-run. (2) Due to the limitations of development conditions in the Tibet region, and considering that the sample from this region might exert extreme effects on the study, we excluded the Tibet samples and re-conducted the regression analysis. display the results of the regression analyses after sample reduction. The regression coefficients for the independent variable digital transformation (DT) on the dependent variable tax avoidance (BTD) were significantly negative at the 10% and 5% statistical levels, respectively. These results indicate that the impact of corporate digital transformation on tax avoidance did not fundamentally change after adjusting the sample period, thus validating the accuracy of the study’s conclusions.

Third, changing the sample scope. Due to the generally higher R D investments in high-tech enterprises, possessing a more substantial foundation and greater potential for digital transformation, this study excludes high-tech enterprises and conducts a new regression analysis. The results in column (6) of Table 7 demonstrate that even after excluding high-tech enterprise samples, digital transformation (DT) continues to significantly reduce corporate tax avoidance behavior, with the regression coefficient of DT on BTD being -0.005, which is significant at the 1% level.

Fourth, extending the observation window. Given that corporate digital transformation is a long-term process that may not manifest results in the short term, the data are regressed again using the independent variable (DT) lagged by two periods. The results in column (1) of Table 7 indicate that the regression coefficient for digital transformation (DT) is -0.003, which is statistically significant at the 5% level. This further corroborates the robustness of the previous research conclusions.

## 5 Further discussion

### 5.1 Mechanism identification and validation

#### A. Technology level

Previous analysis indicates that digital transformation, by leveraging the effects of data elements and information processing, not only provides additional support for corporate RD innovation (Dang et al. 2021) [64], thereby encouraging increased R D investment, but also enhances the integration of production factor resources (Zhang et al. 2021) [75], improving resource allocation efficiency. Consequently, this also results in increased cash flow, reduced risk, and a decrease in tax avoidance behaviors (Rego et al. 2012; Restuccia 2017) [5, 77]. Therefore, this study further constructs Models (2) and (3) to investigate the impact of digital transformation on innovation (RD) and resource allocation efficiency (RAE).

$$\begin{array}{c} {\text{RD}}_{i,t}=\beta_{0}+\beta_{1}DT_{i,t}+\sum_{i=1}^{n}Controls_{i,t}+{\text{Firm}}_{i}+Year_{t}+City_{c}+Year_{t}*Industry_{h}+\epsilon_{i,t} \end{array}$$ (2)

$$\begin{array}{c} {\text{RAE}}_{i,t}=\beta_{0}+\beta_{1}DT_{i,t}+\sum_{i=1}^{n}Controls_{i,t}+{\text{Firm}}_{i}+Year_{t}+City_{c}+Year_{t}*Industry_{h}+\epsilon_{i,t} \end{array}$$ (3)

In Model (2), RD_i,t_ represents innovation, measured as the ratio of annual R D expenditures to total assets, following the approach of most existing studies. The definitions of the other variables are consistent with those in Model (1). In Model (3), RAE_i,t_ represents resource allocation efficiency. This study adopts the measurement method proposed by Richardson (2006) [91], using the absolute value of the residuals calculated by the model to measure RAE, with larger values indicating poorer resource allocation efficiency. The definitions of the other variables still remain consistent with those in Model (1). The specific calculation method is detailed in Eq (4), which first calculates the reasonable investment level for the firm in the current year, followed by the calculation of over-investment to measure the firm’s resource allocation efficiency.

$$\begin{array}{c} {\text{INVEST}}_{n,i,t}=\beta_{0}+\beta_{1}GROWTH_{n,i,t}+\beta_{2}LEV_{n,i,t}+\beta_{3}ROA_{n,i,t}+\beta_{4}AGE_{n,i,t}+\beta_{5}SIZE_{n,i,t}\\ +\beta_{6}INVEST_{n,i,t-1}+Firm_{i}+Year_{t}+\epsilon_{n,i,t} \end{array}$$ (4)

In Eq (4), INVEST_n,i,t_ (fixed asset investment) is the proportion of the original value of fixed assets to the total assets at the beginning of the period; ROA_n,i,t_ is the return on total assets. INVEST_n,i,t-1_ is the firm’s fixed asset investment of the enterprise in year t−1, calculated in the same manner. The definitions of other variables are the same as in the previous text.

Columns (1) and (2) of Table 8 illustrate the validity of the path “digital transformation—technology level change—tax avoidance.” The results show that the regression coefficients for digital transformation on RD and RAE are 0.001 and 0.012, respectively, which are statistically significant at the 5% and 1% levels. This indicates that digitalization significantly enhances the effects of data factors and information processing. Digital transformation can increase liquid cash and mitigate risks through fostering technology level changes, thereby reducing aggressive tax avoidance behavior.

**Table 8:** Regression results of mechanism analysis.

	Technology level		Organisation level	Enviroment level
	(1)	(2)	(3)	(4)
	RD	RAE	IC	UNC_ENV
*DT*	0.001**	0.012***	0.129*	-0.010**
	(2.20)	(3.15)	(1.90)	(-2.54)
*Control variables*	Yes	Yes	Yes	Yes
*Firm effect*	Yes	Yes	Yes	Yes
*Year effect*	Yes	Yes	Yes	Yes
*City effect*	Yes	Yes	Yes	Yes
*Year* − *Industry effect*	Yes	Yes	Yes	Yes
*Obs*	30,405	27,330	22,442	30,377
*adj*.*R*^2^	0.786	0.561	0.118	0.679

Note: (1) ***, **, and * indicate significant at the 1%, 5%, and 10% levels, respectively. (2) Parentheses indicate t-values adjusted by clustering robust standard errors.

#### B. Organisation level

Previous theoretical analysis indicates that digital transformation can enhances internal control quality by improving information transparency and strengthening information integration (Wu 2023) [13]. This, in turn, enhances oversight and management of corporate taxation, reduces managerial opportunism, and mitigates the risk of corporate tax avoidance (Chen et al. 2015; Qi et al. 2020) [12, 79]. Therefore, this study further constructs Model (5) to investigate the impact of digital transformation on internal control quality (IC).

$$\begin{array}{c} {\text{IC}}_{i,t}=\beta_{0}+\beta_{1}DT_{i,t}+\sum_{i=1}^{n}Controls_{i,t}+{\text{Firm}}_{i}+Year_{t}+City_{c}+Year_{t}*Industry_{h}+\epsilon_{i,t} \end{array}$$ (5)

In model (5), IC_i,t_ represents internal control quality, measured using the DiBo internal control index rating. DiBo constructs an index reflecting internal control effectiveness based on the achievement of internal control objectives and classifies it into eight levels from high to low. Therefore, the internal control index rating is used as a proxy variable for measuring internal control effectiveness, with values assigned from 1 to 8, corresponding to low to high levels. The definitions of the other variables are consistent with those in Model (1). The results in column (3) of Table 8 indicate that the regression coefficient of digital transformation (DT) on internal control quality (IC) is significantly positive at the 10% statistical level. This suggests that digital transformation significantly enhances internal control quality and improves corporate governance effectiveness. Consequently, the path of ‘digital transformation—organization level changes—tax avoidance’ is validated.

#### C. Enviroment level

Building on the previous analysis, digital transformation can reduce environmental uncertainty by enhancing information transparency and connectivity (Qi et al. 2020) [12], thereby improving corporate compliance (Sun et al. 83) and reducing tax avoidance behaviors. Therefore, this study further constructs Model (6) to investigate the impact of digital transformation on environmental uncertainty (UNC_ENV).

$$\begin{array}{c} {\text{UNC\_ENV}}_{i,t}=\beta_{0}+\beta_{1}DT_{i,t}+\sum_{i=1}^{n}Controls_{i,t}+{\text{Firm}}_{i}+Year_{t}+City_{c}+Year_{t}*Industry_{h}+\epsilon_{i,t} \end{array}$$ (6)

In model (6), UNC_ENV_i,t_ represents environmental uncertainty, which is reflected in this study through the level of market competition. Specifically, market competition is measured using the Herfindahl-Hirschman Index (HHI), with the index calculated based on firm asset size. A higher HHI value indicates weaker market competition, while a lower HHI value signifies more intense competition. The definitions of the other variables are consistent with those in Model (1). The results in column (4) of Table 8 show that the regression coefficient for digital transformation (DT) on environmental uncertainty (UNC_ENV) is significantly negative at the 5% statistical level. This indicates that an increase in digital transformation significantly reduces corporate environmental uncertainty. Therefore, the path of ‘digital transformation—environment changes—tax avoidance’ is validated.

### 5.2 Heterogeneity analysis

#### 5.2.1 Heterogeneity in the corporate life cycle

According to corporate life cycle theory, significant differences in business decisions such as the degree of digital transformation and tax avoidance behavior may arise across various stages of a company’s development. Digital transformation often entails high costs and risks (Philippart 2021) [92]. Although firms in the growth stage may have relatively immature profitability and operational capabilities, they generally have significant growth potential and a strong willingness to assume risks. They can more easily to meet economic development demands by actively pursuing digital transformation, secure external funding such as government support, and enhance cash flow (Shang et al. 2023) [93], which can reduce tax avoidance behaviors (Frischmann et al. 2008) [51]. In the mature stage, the management structure and economic foundation of enterprises are relatively stable. The impact of digital transformation on altering management structures and enhancing economic foundations is not significant, and there are fewer motivations and opportunities for management to engage in tax avoidance. Based on this analysis and referencing Dickinson (2011) [94], this study categorizes the corporate life cycle into growth, maturity, and decline stages using a composite cash flow approach, and conducts regression analyses to explore the differential impact of digital transformation on tax avoidance across these stages. Specifically, the study utilizes the composite characteristics of net cash flows from operating, investing, and financing activities to measure the life cycle stages of companies (Dickinson 2011) [94]. The columns (1) to (3) of Table 9, reports the regression results based on life cycle heterogeneity. The results indicate that for enterprises in the growth stage, the regression coefficient of digital transformation (DT) on tax avoidance behavior (BTD) is significantly negative at the 5% statistical level. However, for enterprises in the mature and decline stages, the regression coefficient of DT on BTD is not significant. This suggests that digital transformation significantly suppresses tax avoidance behavior in enterprises during the growth stage, which is consistent with theoretical analysis.

**Table 9:** Regression results for heterogeneity in corporate life cycle.

	(1)	(2)	(3)
	BTD	BTD	BTD
	Growth period	Mature period	Decline period
*DT*	-0.003**	-0.00002	-0.002
	(-2.17)	(-0.01)	(-0.83)
*Controlv ariables*	Yes	Yes	Yes
*Firm effect*	Yes	Yes	Yes
*Year effect*	Yes	Yes	Yes
*City effect*	Yes	Yes	Yes
*Year* − *Industry effect*	Yes	Yes	Yes
*Obs*	12,566	11,101	4,406
*adj*.*R*^2^	0.447	0.452	0.416

Note: (1) *** indicate significant at the 1% levels. (2) Parentheses indicate t-values adjusted by clustering robust standard errors.

#### 5.2.2 Heterogeneity in the financing environment

Digital transformation requires sufficient cash flow. Currently, most enterprises are constrained by financing limitations and remain at the preliminary stage of digital transformation (Wang et al. 2022) [95]. The greater the financing pressure faced by enterprises, the poorer the outcomes of digital transformation, which, instead, fails to bring about improvements in economic benefits and management quality. In such cases, management has a strong incentive to engage in earnings manipulation and tax avoidance to maximize private profits. Therefore, the suppressive effect of digital transformation on tax avoidance may be less noticeable for enterprises with higher financing constraints. To investigate the heterogeneity of the effect of digital transformation on tax avoidance in high versus low financing constraint groups, this study performs grouped regressions by dividing enterprises into high and low financing constraint groups based on whether their financing constraint SA index is above or below the median. While various methods for measuring financing constraints exist, the SA index effectively avoids endogenous interference and is computationally convenient and relatively robust (Ju et al. 2013) [96]. Following Hadlock and Pierce (2010) [97], the SA index is constructed using only two variables: firm size and firm age, which exhibit minimal temporal variation and strong exogeneity. Specifically, the SA index is calculated as -0. 737*SIZE + 0.043*SIZE2—0.04*AGE, where SIZE denotes the natural logarithm of total assets at year-end, and AGE is the difference between the current year and the year the firm was listed. Table 10 Columns (1)-(2) display the regression results for financing environment heterogeneity. The results indicate that when enterprises face lower financing constraints, digital transformation enable significantly suppresses tax avoidance behavior. This is because lower financing constraints allow enterprises to reduce financial pressure through internal and external funding support, further mitigating the negative impacts of the high costs and risks associated with digital transformation, optimizing resource allocation, and thus reducing the motivation and behavior of tax avoidance.

**Table 10:** Financing environment and regional heterogeneity regression results.

	Financing constraints:High	Financing constraints:Low	East	Mid	West
	(1)	(2)	(3)	(4)	(5)
	BTD	BTD	BTD	BTD	BTD
*DT*	-0.002	-0.003*	-0.002*	-0.005*	0.001
	(-1.34)	(-1.86)	(-1.80)	(-1.89)	(0.27)
*Control variables*	Yes	Yes	Yes	Yes	Yes
*Firm effect*	Yes	Yes	Yes	Yes	Yes
*Year effect*	Yes	Yes	Yes	Yes	Yes
*City effect*	Yes	Yes	Yes	Yes	Yes
*Year* − *Industry effect*	Yes	Yes	Yes	Yes	Yes
*Obs*	14,904	15,113	21,319	4,132	4,939
*adj*.*R*^2^	0.435	0.461	0.428	0.407	0.495

Note: (1)* indicate significant at the 10% level. (2) Parentheses indicate t-values adjusted by clustering robust standard errors.

#### 5.2.3 Heterogeneity of regions

Compared to the western region, the eastern and central regions exhibit a relatively higher degree of marketization, faster information flow, lower financing pressure, and richer financial resources. Consequently, the digital transformation process is more advanced, and the level of digital development is relatively higher in these regions. This high level of digital development provides more convenient and efficient channels for information exchange between firms and tax authorities. Tax authorities can utilize digital tools to more precisely monitor firms’ tax situations, reducing the likelihood of tax avoidance. In contrast, the western region, due to relatively lagging economic development, has a slower digital transformation process and weaker regulatory capabilities, potentially allowing more room for tax avoidance. Therefore, this study infers that the effect of digital transformation on curbing tax avoidance varies by region and may be more pronounced in the eastern and central regions. To this end, following the approach of most existing studies, provinces are categorized into eastern, central, and western regions for regression analysis, as shown in columns (3) to (5) of Table 10. In the eastern and central regions, the regression coefficients of digital transformation (DT) on corporate tax avoidance (BTD) are -0.002 and -0.005, respectively, and are significant at the 10% statistical level. This indicates that in these regions, enhancing digital transformation effectively reduces tax avoidance behavior. However, in the western region, the regression coefficient of DT on BTD does not pass the statistical significance test. This findings indicates a regional disparity in the effectiveness of digital transformation in curbing tax avoidance (Wang et al. 2023) [98]. Additionally, the regression coefficient of DT on BTD is higher in the central region compared to the eastern region, suggesting that digital transformation in the central region has a stronger effect on suppressing tax avoidance. In recent years, the central region has been progressively evolving an industrial model dominated by emerging digital and smart manufacturing industries as it absorbs industrial transfer from the eastern coastal regions. Consequently, firms in this region have greater advantages and incentives for digital transformation, playing a significant role in enhancing the standardization and transparency of tax administration, thereby also curbing tax avoidance.

### 5.3 Long-term consequences: The economic consequences test

Total factor productivity (TFP) is a critical indicator for evaluating high quality economic development and a primary driver of sustainable economic growth (Young 1996; Lei et al. 2023) [99, 100]. Digital transformation has been shown to enhance total factor productivity either directly (Pan et al. 2022) [101] or indirectly by improving technological innovation and resource allocation efficiency, reducing operational costs, and optimizing human capital structure (Lei et al. 2023) [100]. Crocker and Slemrod (2005) [102] have indicated that improving business efficiency depends on enhancements in tax administration intensity and increased tax compliance by firms. Furthermore, Liu et al. (2019) [103] have also indicated that increased tax avoidance negatively impacts TFP. Therefore, this study futher investigates whether digital transformation can enhance TFP by reducing corporate tax avoidance. To this end, the study employs a stepwise regression approach and constructs the following econometric models for robustness checks, referencing the methods for testing mediation effects proposed by Hayes (2009) [104] and Wen et al. (2014) [105].

$$\begin{array}{c} {\text{TFP}}_{i,t}=\beta_{0}+\beta_{1}DT_{i,t}+\sum_{i=1}^{n}Controls_{i,t}+{\text{Firm}}_{i}+Year_{t}+City_{c}+Year_{t}*Industry_{h}+\epsilon_{i,t} \end{array}$$ (7)

$$\begin{array}{c} {\text{BTD}}_{i,t}=\beta_{0}+\beta_{1}DT_{i,t}+\sum_{i=1}^{n}Controls_{i,t}+{\text{Firm}}_{i}+Year_{t}+City_{c}+Year_{t}*Industry_{h}+\epsilon_{i,t} \end{array}$$ (8)

$$\begin{array}{c} {\text{TFP}}_{i,t}=\beta_{0}+\beta_{1}BTD_{i,t}+\beta_{2}DT_{i,t}+\sum_{i=1}^{n}Controls_{i,t}+{\text{Firm}}_{i}+Year_{t}+City_{c}+Year_{t}*Industry_{h}+\epsilon_{i,t} \end{array}$$ (9)

Model (8) is consistent with model (1). In models (7) and (9), TFP denotes total factor productivity. Currently, the semi-parametric estimation methods proposed by Olley and Pakes (1996) and Levinsohn and Petrin (2003) are commonly used to measure total factor productivity (Lei et al. 2023) [100]. However, the Olley-Pakes method requires a strict monotonic relationship between inputs and outputs, which means that samples with zero investment cannot be estimated. Consequently, this study adopts the Levinsohn-Petrin method to measure total factor productivity to address the limitations of the Olley-Pakes method. Aside from this, the definitions of other variables remain consistent with those in previous sections. This section primarily examines the regression coefficients of DT and BTD on TFP to assess whether the path “digital transformation—corporate tax avoidance—total factor productivity” holds.

The results in column (1) of Table 11 show that the regression coefficient of DT on TEP is 0.039, which is statistically significant at the 1% level, indicating that digital transformation significantly enhances total factor productivity. The results in column (2) are consistent with the baseline regression results in Table 4, demonstrating that digital transformation significantly reduces corporate tax avoidance. In column (3), the regression coefficient of DT on TFP is 0.037, while BTD’s coefficient on TFP is -0.926, both of which are statistically significant at the 10% and 1% levels, respectively, aligning with theoretical expectations. Additionally, the Sobel test for mediation effects yields a Z-statistic of 2.111, which is significant at the 5% level, confirming the presence of a mediation effect. Furthermore, the decrease in the regression coefficient of DT in column (3) compared to column (1) suggests that the occurrence of tax avoidance weakens the “dividend” effect of digital transformation on total factor productivity. This further confirms that the path of ‘digital transformation—corporate tax avoidance—total factor productivity’ is validated. In summary, the results confirm that the main conclusion of this study-that digital transformation’s capacity to reduce tax evasion can further improve firm productivity-is valid.

**Table 11:** Long-term economic consequence test results.

	(1)	(2)	(3)
	TFP	BTD	TFP
*DT*	0.039*	-0.002**	0.037*
	(1.95)	(-2.09)	(1.84)
*BTD*			-0.926***
			(-9.27)
*Control variables*	Yes	Yes	Yes
*Firm effect*	Yes	Yes	Yes
*Year effect*	Yes	Yes	Yes
*City effect*	Yes	Yes	Yes
*Year* − *Industry effect*	Yes	Yes	Yes
*Obs*	28,289	30,405	28,289
*adj*.*R*^2^	0.932	0.434	0.932
*Sobeltest*		**Mediator variable:BTD**	
		2.111**	
*Ind*_*e*_*fftest*(*P* − *val*)		**The mechanism is effective**	
		**0.035**	
		**The indirect effect holds**	

Note: (1) (1) **, and * indicate significant at the 5% and 10% levels, respectively. (2) Parentheses indicate t-values adjusted by clustering robust standard errors.

## 6 Fundamental assurance for the effectiveness of digital transformation: A perspective based on business risks

At present, both academics and practitioners are actively exploring optimization paths for tax governance in the digital economy era. Previous theoretical and empirical studies have demonstrated that digital transformation can significantly curb corporate tax avoidance and that this effect varies based on corporate attributes, industry environment, and regional development. To maximize the impact of digital transformation on curbing corporate tax avoidance and thereby provide a basis for reducing national tax revenue loss and optimizing tax governance, this study builds upon previous research to further investigate the fundamental assurance necessary for the effectiveness of digital transformation from an business risk perspective.

In the digital economy era, the updating and iteration of technology, improvements in government policy, and international environmental turbulence have led to increased business risks, exacerbating uncertainties in production and operations(Wang et al. 2017) [106], Consequently, these factors may also influence the formulation and effectiveness of digital transformation decisions. This study posits that as the level of operational risk increases, management may redirect more attention and resources towards risk management and control. They may even employ tax avoidance strategies to generate revenue, thereby balancing the impact of business risk on the company’s production and business activities, futher reducing engagement in high-cost, high-risk activities like digital transformation. Additionally, an increased level of business risk may impair financial performance (Horcher 2010; Manuj 2014) [107, 108], affect resource allocation, trigger ‘precautionary savings’ motivations (He et al 2022) [109], reduce the benefits of high-cost activities (Wang et al. 2017; Zhu et al. 2020) [106, 110], and prevent the adverse effects of cash flow uncertainty on business operations (Wang et al. 2019) [111]. While digital transformation can enhance organizational efficiency (Loebbecke et al. 2015) [43] and reduce costs, it may not immediately translate into cash flow in the short term. Consequently, managers may seek other business activities to increase cash flow to alleviate financial pressure. Additionally, an increase in business risk may challenge the company’s internal control systems, raising the likelihood of non-compliance and creating conditions conducive to tax avoidance, which can hinder efforts to curb tax avoidance. Therefore, the stability of the external environment will be fundamental assurance to the effectiveness of digital transformation. Based on the above analysis, this study aims to integrate business risk endowment conditions into the “digital transformation—tax avoidance” research paradigm to examine how digital transformation affects tax avoidance under varying levels of business risk.

This study characterizes business risk through the degree of volatility in corporate profits, following the approach of Wang et al. (2017) [106]. Research by John et al. (2008) and Acharya et al. (2011) [112, 113] both suggest that higher business risk is associated with increased profits volatility. Detailed calculations are provided in Eq (10).

$$\begin{array}{c} \Delta_{i,t}={\sqrt{\frac{T}{T-1}\sum_{i=1}^{n}\left(E_{i,t}-\frac{1}{T}\sum_{i=1}^{n}E_{i,t}\right)}}^{2}|T=4,E_{i,t}=\frac{EBIT_{i,t}}{A_{i,t-1}} \end{array}$$ (10)

In Eq (10), Δ_i,t_ represents the business risk of firm i in year t; EBIT_i,t_ denotes the earnings before interest, taxes, depreciation, and amortization (EBITDA) of firm i in year t; and A_i,t_ signifies the total assets of firm i in year t-1. As illustrated in the formula, business risk is calculated using the standard deviation of the rolling EBITDA margin value from years t-4 to t-1. Additionally, since the business risk calculated using this method does not follow a normal distribution, this study computes the cumulative distribution probability of the EBITDA margin’s standard deviation to measure business risk (RISK).

This study first investigates the core influence of business risk levels on digital transformation. Considering the long-term effects of business risk, a two-period lag in business risk levels is used for the regression analysis. Subsequently, the sample is divided into high and low business risk groups based on the median, and the core effect of ‘digital transformation-tax avoidance’ is re-examined through regression. This approach aims to explore the differentiated impact of digital transformation on tax avoidance under varying levels of operational risk, as illustrated by the results in Table 12.

**Table 12:** Regression results from a business risk perspective.

	(1)	(2)	(3)
	DT	BTD	BTD
		Business risk: High	Business risk: Low
*L*2.*RISK*	-0.002*		
	(-1.94)		
*DT*		-0.007	-0.020***
		(-1.32)	(-3.75)
*Control variables*	Yes	Yes	Yes
*Firm effect*	Yes	Yes	Yes
*Year effect*	Yes	Yes	Yes
*City effect*	Yes	Yes	Yes
*Year* − *Industry effect*	Yes	Yes	Yes
*Obs*	15,016	11,189	10,762
*adj*.*R*^2^	0.630	0.433	0.471

Note: (1) (1) **, and * indicate significant at the 5% and 10% levels, respectively. (2) Parentheses indicate t-values adjusted by clustering robust standard errors.

The results in column (1) of Table 12 how that the regression coefficient of business risk level (L2.RISK) on digital transformation (DT) is significantly negative at the 10% statistical level, indicating that higher business risk levels significantly inhibit digital transformation. Columns (2) and (3) of Table 12 report the suppressive effects of digital transformation on tax avoidance under high and low business risk levels, respectively. The results reveal that lower business risk levels futher enhance the effectiveness of digital transformation in curbing tax avoidance. A reduction in operational risk levels can enhance financial performancereport the suppressive effects of digital transformation on tax avoidance under high and low business risk levels, respectively. The results reveal that lower business risk levels futher enhance the effectiveness of digital transformation in curbing tax avoidance. A reduction in operational risk levels can enhance financial performance (Horcher 2010; Manuj 2014) [107, 108], optimize resource allocation, decrease the motivation for “precautionary savings” (He et al. 2022) [109], and improve the management’s risk control and response capabilities. This, in turn, creates “facilitation” for digital transformation, ensuring its effective implementation, reduces corporate tax avoidance, and thereby provides support for promoting the deep integration of the digital economy with the real economy and optimizing tax governance in the digital era.

## 7 Research conclusions and recommendations

### 7.1 Research conclusions

The widespread nature of corporate tax avoidance (Hanlon et al. 2010) [4] remains a significant impediment to economic development in the digital economy era. However, the prominence in the role of data also presents severe challenges to traditional economic paradigms (Stiglitz 2002) [1], leading to a series of tax administration issues (Huang 2023) [10]. Digital transformation has become an essential skill for business development in the digital economy era. Therefore, reducing corporate tax avoidance through digital transformation, promoting the deep integration of the digital economy with the real economy, and optimizing tax governance in the digital economy era are particularly important. To this end, this study uses a sample of all A-share listed companies from 2007 to 2022, employing a systemic perspective and drawing on signaling theory, information asymmetry theory, and the T-O-E framework to investigate the core impacts of digital transformation on corporate tax avoidance, the intermediary mechanisms involved, and the foundational assurance for effective implementation. Theoretical and empirical analysis yield the following conclusions. (1) The implementation of digital transformation can significantly inhibit corporate tax avoidance, and this conclusion remains valid after a series of robustness tests. This finding is consistent with the results of Zhang et al. (2022) [17] and Xie et al. (2023) [114]. Furthermore, building on this, we also demonstrate that digital transformation can enhance corporate production efficiency by curbing tax avoidance. (2) Digital transformation induces a comprehensive organizational change from top to bottom (Vial 2019) [52], which also triggers multi-dimensional transformations across technology, organization, and environment. Specifically, digital transformation can reduce corporate tax avoidance by improving innovation and resource allocation efficiency (technology level), enhancing internal control quality (organization level) (Guo et al. 2023; Zhou et al. 2022) [19, 20], and lowering industry competition (environment level). (3) The effect of digital transformation on curbing tax avoidance is more pronounced in firms that are in a growth phase, face lower financing constraints, and are located in the central and western regions. (4) Lower business risk is a fundamental assurance for maximizing the effectiveness of digital transformation and curbing corporate tax avoidance.

### 7.2 Research recommendations

#### 7.2.1 Government perspective

(1) The vigorous development of the digital economy has presented numerous challenges to government tax administration. To safeguard national tax revenue and reduce the loss of government tax income, tax authorities should intensify efforts to enhance digital transformation infrastructure and support, decrease the level of information asymmetry between tax authorities and businesses, and strengthen information flow between both parties (Pomeranz 2015) [57], and thereby achieving effective oversight of corporate tax avoidance. Simultaneously, it is crucial to improve relevant laws and regulations considering the unique characteristics of data elements and to strengthen the tax administration and governance system in the digital economy era (Chen et al. 2023) [26].

(2) Tax enforcement intensity and environmental uncertainty are significant factors influencing corporate tax avoidance (Zhou et al. 2022) [20]. The heterogeneity analysis in this study also confirms that an intensified industry competition environment increases corporate tax avoidance behavior. Therefore, the government, as the “visible hand,” should further enhance efforts to foster a fair competitive market environment, reduce uncertainty, and play a crucial role in guiding the healthy and orderly development of the economy. Additionally, attention should also be given to the development status of enterprises in the central and western regions, strengthening digital transformation efforts in the central region to accelerate the digital transformation of high-potential enterprises in the central region, and stimulating the emergence of new digital transformation forces in the western region to reduce regional disparities in digital transformation development (Wang et al. 2023) [98].

#### 7.2.2 Enterprise perspective

(1) In the context of digital economy development, enterprises should first actively respond to government calls for digital transformation, strengthen digital transformation efforts, and vigorously promote the application of digital technologies such as big data, artificial intelligence, and blockchain. They should fully address the technological and organizational changes triggered by digital transformation, curb managerial motives for tax avoidance and rent-seeking, alleviate principal-agent issues (Wang et al. 2023) [2], enhance governance effectiveness, and establish internal control mechanisms aligned with business development objectives (Wu 2023) [13]. Additionally, enterprises should leverage digital technologies to improve information acquisition and processing capabilities (Loebbecke et al. 2015) [43], reduce information gaps, empower innovation through digital transformation, enhance resource allocation capabilities, minimize the losses of economic benefits (Restuccia 2017) [77], and reduce the occurrence of tax avoidance behaviors that operate on the fringes of legality. Concurrently, digital transformation decisions should be adjusted in accordance with the development status of the enterprise at different lifecycle stages to fully realize the empowering effects of digital transformation.

(2) Enterprises should enhance their risk management capabilities to effectively address the uncertainties arising from financing constraints and business risks. Due to financing constraints, most enterprises currently remain at the early stages of digital transformation (Wang et al. 2022) [95]. The greater the financing constraints, the poorer the digital transformation outcomes. Additionally, the presence of business risks introduces uncertainty into a company’s cash flow, which adversely affects its operations (Wang et al. 2019) [111] thereby impeding the effectiveness of digital transformation. Therefore, enterprises should futher enhance their risk management capabilities by establishing robust risk management systems and procedures, identifying, assessing, and monitoring potential risks, and formulating effective risk response strategies to promptly address market, financial, and business risks during their business operations, thereby better meeting regulatory requirements and avoiding impediments to sustained development due to non-compliance issues.

### 7.3 Research limitations and prospects

Although this study aims to analyze the impact of digital transformation on corporate tax avoidance from a systematic perspective, it still has certain limitations. Firstly, the research sample is primarily limited to all A-share listed companies, and has yet to further investigate how digital transformation impacts tax avoidance across different enterprise classifications. Secondly, the analysis is mainly focused on corporate tax avoidance under income tax. However, given the diversity of tax types in China, corporate tax avoidance behavior varies under different tax policies and measurement standards. Therefore, future research could delve into the relationship between digital transformation and tax avoidance by examining various tax types and measurement standards for tax avoidance, thereby providing additional theoretical and empirical evidence.
